# Optimal resource allocation with spatiotemporal transmission discovery for effective disease control

**DOI:** 10.1186/s40249-022-00957-1

**Published:** 2022-03-25

**Authors:** Jinfu Ren, Mutong Liu, Yang Liu, Jiming Liu

**Affiliations:** Department of Computer Science, Hong Kong Baptist University, Kowloon, Hong Kong Special Administrative Region People’s Republic of China

**Keywords:** COVID-19, Omicron outbreak, Densely populated regions, Spatiotemporal transmission risk, Optimal resource allocation, Integrative case management, Effective disease control

## Abstract

**Background:**

The new waves of COVID-19 outbreaks caused by the SARS-CoV-2 Omicron variant are developing rapidly and getting out of control around the world, especially in highly populated regions. The healthcare capacity (especially the testing resources, vaccination coverage, and hospital capacity) is becoming extremely insufficient as the demand will far exceed the supply. To address this time-critical issue, we need to answer a key question: *How can we effectively infer the daily transmission risks in different districts using machine learning methods and thus lay out the corresponding resource prioritization strategies, so as to alleviate the impact of the Omicron outbreaks?*

**Methods:**

We propose a computational method for future risk mapping and optimal resource allocation based on the quantitative characterization of spatiotemporal transmission patterns of the Omicron variant. We collect the publicly available data from the official website of the Hong Kong Special Administrative Region (HKSAR) Government and the study period in this paper is from December 27, 2021 to July 17, 2022 (including a period for future prediction). First, we construct the spatiotemporal transmission intensity matrices across different districts based on infection case records. With the constructed cross-district transmission matrices, we forecast the future risks of various locations daily by means of the Gaussian process. Finally, we develop a transmission-guided resource prioritization strategy that enables effective control of Omicron outbreaks under limited capacity.

**Results:**

We conduct a comprehensive investigation of risk mapping and resource allocation in Hong Kong, China. The maps of the district-level transmission risks clearly demonstrate the irregular and spatiotemporal varying patterns of the risks, making it difficult for the public health authority to foresee the outbreaks and plan the responses accordingly. With the guidance of the inferred transmission risks, the developed prioritization strategy enables the optimal testing resource allocation for integrative case management (including case detection, quarantine, and further treatment), i.e., with the 300,000 testing capacity per day; it could reduce the infection peak by 87.1% compared with the population-based allocation strategy (case number reduces from 20,860 to 2689) and by 24.2% compared with the case-based strategy (case number reduces from 3547 to 2689), significantly alleviating the burden of the healthcare system.

**Conclusions:**

Computationally characterizing spatiotemporal transmission patterns allows for the effective risk mapping and resource prioritization; such adaptive strategies are of critical importance in achieving timely outbreak control under insufficient capacity. The proposed method can help guide public-health responses not only to the Omicron outbreaks but also to the potential future outbreaks caused by other new variants. Moreover, the investigation conducted in Hong Kong, China provides useful suggestions on how to achieve effective disease control with insufficient capacity in other highly populated countries and regions.

**Graphical Abstract:**

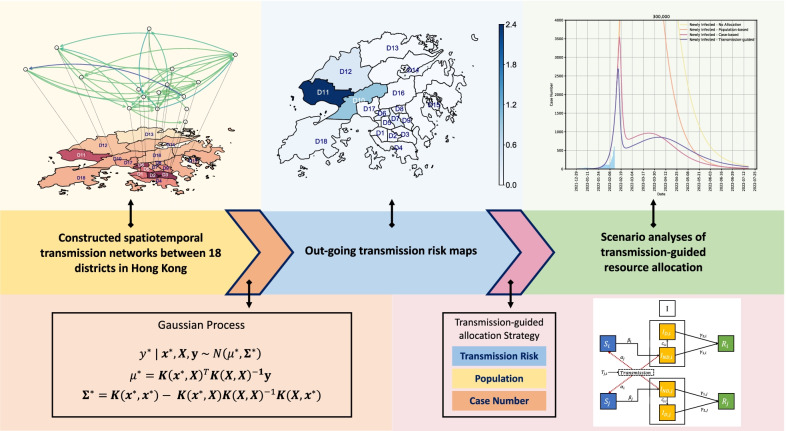

**Supplementary Information:**

The online version contains supplementary material available at 10.1186/s40249-022-00957-1.

## Background

The emergence of the SARS-CoV-2 Omicron variant (B.1.1.529) posts an unprecedented challenge to the control and prevention of the pandemic of COVID-19 [1–4]. Studies show that the Omicron variant is four times as transmissible as the Delta variant [5–7], and it is now dominating the transmission in the current pandemic. The healthcare system is now suffering from a tremendous pressure of the capacity shortage (especially the shortage in testing resources, vaccination coverage, and hospital capacity) due to the extremely strong transmissibility of the Omicron variant [8], especially in the densely populated regions [9, 10]. Take Hong Kong of China, a metropolis with a total population size of 7.5 million and a population density of 7140 per square kilometre, as an example. The current daily testing capacity in Hong Kong, China is far less than 300,000 [11, 12]. Compared with the total population, such a capacity is obvious insufficient for detecting the infected cases and containing the disease transmission, especially when it is not optimally directed to the communities with the highest risk or the greatest need [13, 14].

To optimally allocate the healthcare resources to mitigate the spread of the epidemic, especially when the resources are limited, a critical question therefore needs to be answered: *How can we effectively infer the daily transmission risks in different districts using machine learning methods and thus lay out the corresponding resource prioritization strategies, so as to alleviate the impact of the Omicron outbreaks?* To answer this question, we must address two technical challenges:

1. How can we foresee the future risks of the Omicron outbreaks in different locations?
2. How can we allocate the limited healthcare resources based on the future risks to reduce the impact of the outbreak?

To address these two challenging issues, in this study, we propose a computational method for future risk mapping and optimal resource allocation based on the quantitative characterization of spatiotemporal transmission patterns of the Omicron variant. As shown in existing studies, spatiotemporal transmission patterns of the virus play a key role in uncovering the dynamism of the disease spread and outbreaks [15–18], and thus should be taken into account when investigating the disease transmission risks and strategizing the corresponding interventions. Specifically, we design our method as follows. First, we construct the spatiotemporal transmission matrices across different districts based on the publicly available records of confirmed cases, which contains the visiting history of each case. With the constructed cross-district transmission matrices, we then forecast the future risks of various locations daily by means of the Gaussian process. Finally, we develop a transmission-guided resource prioritization strategy that enables effective control of Omicron outbreaks under limited capacity.

We validate the effectiveness of the proposed method in forecasting the risks and prioritizing the resources through a comprehensive investigation in Hong Kong, China. The maps of district-level transmission risks show clear spatiotemporal heterogeneity, providing key information to resource allocation. With the guidance of the inferred transmission risks, the developed prioritization strategy enables the effective and flexible testing resource allocation with different levels of capacity insufficiency. As shown in this study, the quantitative characterization of the spatiotemporal transmission patterns of the disease enables us to gain the insights into the dynamism and heterogeneity of the outbreaks as well as the implementation and optimization of the prioritized resource allocation strategies. This is not only of critical importance in preventing the Omicron outbreaks in Hong Kong, China, but also informative in guiding the responses to the potential future outbreaks caused by other new variants and/or in other highly populated countries and regions under serious capacity shortage.

## Methods

### Data sources and collection

We develop the method and conduct the investigation based on the information of reported COVID-19 cases in Hong Kong, China, covering all 18 districts from March 17, 2020 to February 5, 2022. The data are owned and publicly released by the Department of Health, Hong Kong Special Administrative Region (HKSAR) Government [19]. For each confirmed case, we use the individual’s onset date, report date, and the buildings that the individual visited during the 14 days before the date of case confirmation to construct the spatiotemporal transmission matrices. We identify the location of the buildings (i.e., their latitudes and longitudes) using the building names from the Google Geocoding API. We further use the constituency area shape file, which contains the latitudes and longitudes of the boundary of constituency areas to identify the constituency areas that have been visited by the confirmed cases.

### Construction of spatiotemporal transmission matrices

We construct each disease transmission matrix (daily) across 18 districts in Hong Kong, China as follows. First, we build the daily transmission matrix at the constituency area level. Specifically, for each confirmed case, if they have visited a series of locations (i.e., buildings) in the same day, we construct a link between any two building *i* and *j*, and the direction of the link is from *i* to *j* if the building *i* appeared before the building *j* in the case-visited building list provided by the government. We take the difference between the day of visit and the symptom onset day (in terms of the number of days) as the input of the infectiousness profile [20] to get the transmission risk from *i* to *j*, which is introduced by the connection between these two buildings. We then identify the corresponding constituency area that each building belongs to, and accumulate the building-to-building transmission risks to form the transmission matrix at the constituency area level. Based on the 452 $\times$ 452 constituency-level matrix (since there are 452 constituency areas in Hong Kong, China), we further construct the 18 $\times$ 18 district-level transmission matrix by summating the elements in the corresponding district block. For example, D5 (the district of Yau Tsim Mong) has 20 constituency areas while D8 (the district of Wong Tai Sin) has 25 constituency areas. As a result, the transmission risk from D5 to D8 will be the summation of all elements in the corresponding 20 $\times$ 25 block. Note that for each day there will be a corresponding transmission matrix across districts. Please refer to the Additional file 1 for more details of the construction of spatiotemporal transmission matrices.

### District-level risk mapping

Based on the constructed daily transmission matrix, we infer the daily transmission risks of different districts as follows. First, we define the daily transmission risk of each district as its transmissibility not only to itself but also to the remaining 17 districts. Therefore, we can obtain such risk of a specific district by summating all elements of the corresponding row in the constructed transmission matrix, in which the (*i*, *j*)-th element denotes the transmission risk from the *i-*th district to the *j*-th district. By doing so, we can obtain a series of daily transmission risks for each district in a given period. Then we infer the transmission risks of different districts by adopting the Gaussian process regression model [21, 22], which is a representative machine learning model for time series prediction with uncertainty. We use the historical data to train the model and then use the trained model to predict the future transmission risks of all districts. Please refer to the Additional file 1 for more details of the procedure of district-level risk inference.

### Transmission-guided resource allocation

**Fig. 1:**
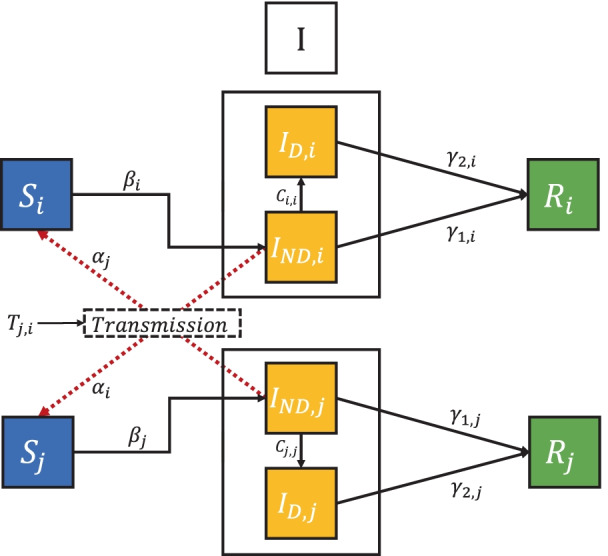
The structure of the proposed SDNR compartmental model

To optimally direct resources to the communities with the greatest needs, we develop a transmission-guided strategy, which prioritizes different districts for testing resource allocation according to their population size, number of infections, and more importantly, the transmission risk. Note that the testing discussed in this study is not an isolated action but an integrative case management process, including testing for detection, quarantine, and further treatment. Therefore, in our study, we consider that the infected individuals detected by the testing will be immediately quarantined and thus cannot further infect other susceptible individuals. To validate the effectiveness of our strategy, we further develop a compartmental model at the meta-population level called Susceptible-Detected-Nondetected-Recovered (SDNR) model to simulate the future trend of the disease transmission. The structure of proposed SDNR model is shown in Fig. 1. In each district, the proposed SDNR model divides the compartment of infectious population into two sub-groups: detected infections and non-detected infections, which are decided by the allocation rate of resources and the infection rate. The 18 individual compartmental models (one for each district) are integrated in our SDNR model using the constructed transmission matrices across 18 districts. Please refer to the Additional file 1 for more details of the transmission-guided resource allocation.

## Results

### Results of the transmission matrix construction

**Fig. 2:**
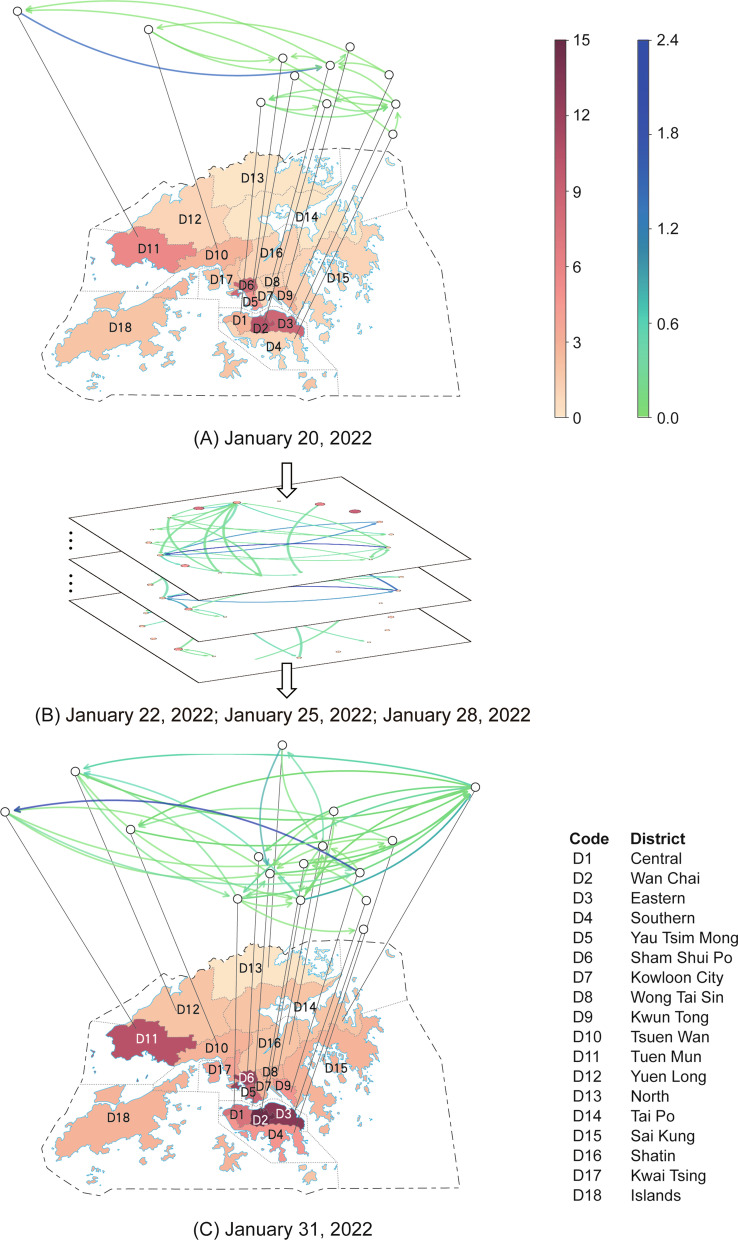
The case maps and the constructed spatiotemporal transmission networks on (**A**) January 20, 2022, (**B**) January 22, 2022, January 25, 2022, and January 28, 2022, and (**C**) January 31, 2022 in Hong Kong, China. The red map at the bottom of (**A**) and (**C**) denotes the case map of the corresponding day. The intensity of the red color indicates the number of the cases. The darker the color on the map, the more cases in the corresponding district. The networks in the blue/green color shown in (**B**) and at the top of (**A**) and (**C**) are the spatiotemporal transmission networks constructed from the case visiting history. The blue color indicates the high transmission intensity while the green color indicates the low intensity. The correspondence between the district code and the district name is provided in the bottom right corner of the figure

We construct the daily spatiotemporal transmission matrices in Hong Kong, China during December 27, 2021 to February 5, 2022, which is corresponding to the current wave of Omicron outbreak in Hong Kong, China. Figure 2 illustrates several examples of the transmission networks reflected by the constructed matrices. The correspondence between the district code and the district name is provided in the bottom right corner of the figure as well as Table S1 of the Additional file 1. The bottom maps in subfigures (A) and (C) demonstrate the number of reported cases in different districts on January 20, 2022 and January 31, 2022, respectively. The network above the corresponding map visualizes the constructed daily transmission matrix across different districts in the same day. In the network, each node denotes the corresponding district below it, connected by the dotted line, and the color of the links indicates the transmission intensity from one district to another. The elements’ values of the constructed transmission matrices on January 20, 2022 and January 31, 2022 are provided in Tables S2 and S3 of the Additional file 1, respectively. Figure 2B shows the transmission networks in other three different days during the period of January 20, 2022–January 31, 2022.

From the figure we can observe that edges in the same transmission network (i.e., the same day) show obvious difference in their intensities, indicating the spatial heterogeneity of the transmission patterns. Moreover, the networks are time-varying, even for two close dates, demonstrating the temporal heterogeneity of the transmission patterns. Such complex spatiotemporal heterogeneity determines the dynamics of the transmission and makes the outbreaks difficult to forecast. By quantitatively characterizing such spatiotemporal transmission patterns, our method can uncover the underlying dynamism of the disease, and thus enables the risk mapping and resource allocation, which will be shown in the following two subsections.

### Results of risk mapping of districts

**Fig. 3:**
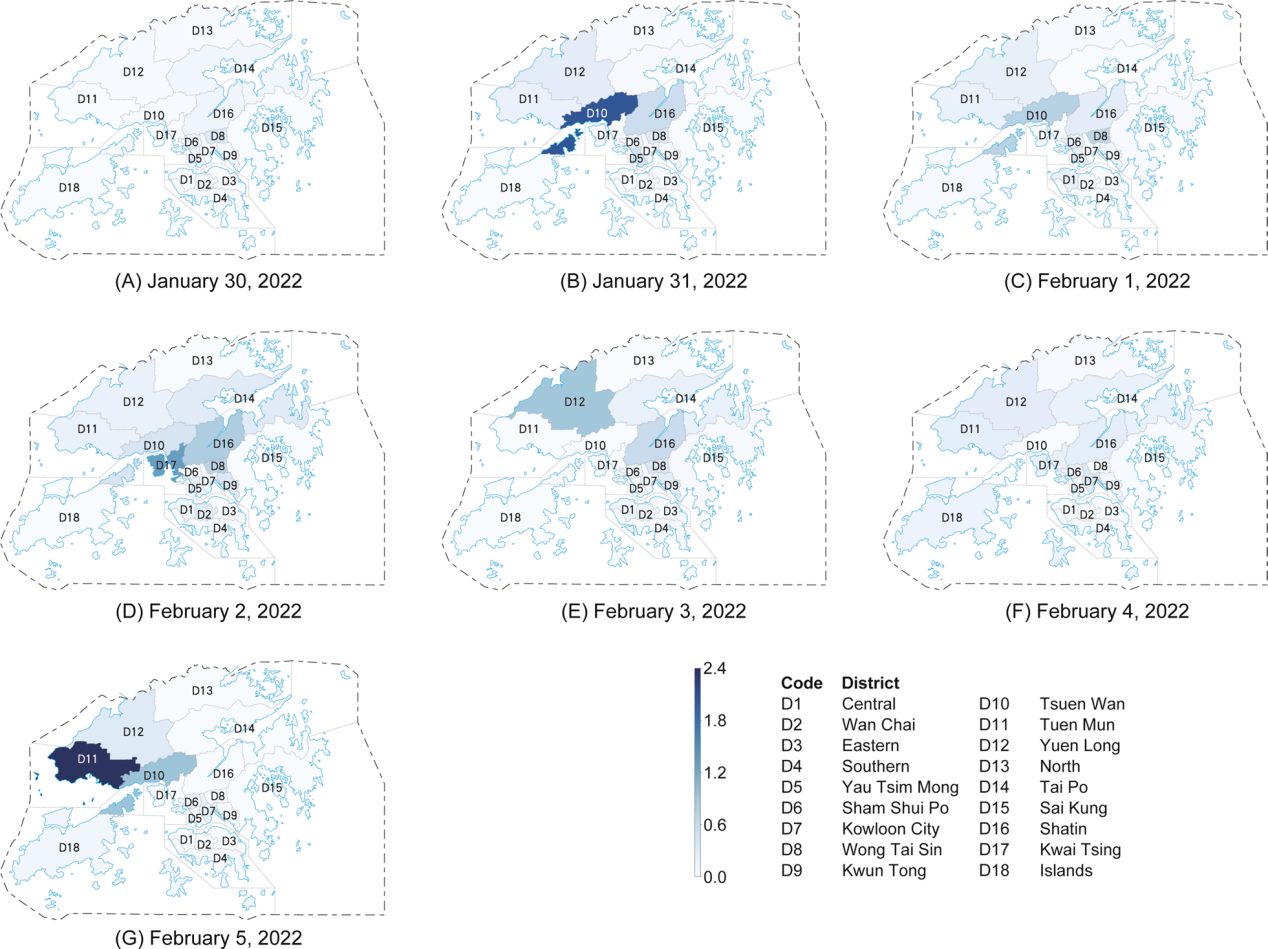
The out-going transmission risk maps of 18 districts in Hong Kong, China from (**A**) January 30, 2022 to (**G**) February 5, 2022. The intensity of the blue color indicates the transmission risk level. The darker the color on the map, the higher the risk of the corresponding district. The correspondence between the district code and the district name is provided in the bottom right corner of the figure

Figure 3 illustrates the transmission risks of all 18 administrative districts in Hong Kong, China from January 30, 2022 to February 05, 2022, which are inferred from the Gaussian process model. Detailed results of inferred risk mapping and complete ranking results of the transmission risks of these districts are provided in Tables S5 and S6 of the Additional file 1, respectively.

Similar to the spatiotemporal heterogeneity of the transmission networks observed in Fig. 2, the transmission risks in different districts also demonstrate obvious heterogeneity in both spatial and temporal dimensions, which can be seen in Fig. 3. From January 30, 2022 to February 05, 2022, the district with the highest transmission risk has changed five times in just one week (D8: Wong Tai Sin on January 30, 2022, D10: Tsuen Wan on January 31, 2022 and February 1, 2022, D17: Kwai Tsing on February 2, 2022, D12: Yuen Long on February 3, 2022, D7: Kowloon City on February 4, 2022, and D11: Tuen Mun on February 5, 2022). This result demonstrates the highly dynamic patterns of the disease transmission risks, implying that purely relying on passively tracing the historical cases might be less effective for outbreak control, especially in such a densely populated and highly dynamic environment.

Another interesting observation is that the districts with more cases may not necessarily indicate the higher transmission risks, and vice versa. For example, on February 2, 2022, the top-3 districts with the highest transmission risks are D17 (Kwai Tsing), D16 (Shatin), and D8 (Wong Tai Sin). In the same day, however, the top-3 districts with the largest number of cases are D2 (Wan Chai), D3 (Eastern), and D6 (Sham Shui Po), which are totally different from the districts with the highest risks. This observation indicates that the number of detected cases might not be the golden criterion to quantify the risks. To provide a more effective characterization, the spatiotemporal transmission patterns should also be taken into consideration, so as to enable the early warning of potential outbreaks and the response planning in advance.

### Scenario analyses of transmission-guided resource allocation

To validate the effectiveness of the transmission-guided strategy for allocating resources, we conduct a series of simulations with different levels of testing resource capacity: 300,000, 500,000 and, 700,000 per day, respectively, which are consistent with the current capacity in Hong Kong or expected to be achieved in the near future with the support from the mainland of China [12]. As mentioned previously, testing here indicates an integrative case management including testing for detection, quarantine, and further treatment. That is to say, the cases detected by the test will be quarantined and treated, and thus will not further infect other people. We consider four scenarios with different resource allocation strategies: (1) Baseline: no specific strategies will be adopted; (2) Allocating the testing resources according to the population size of each district (population-based strategy): this is somewhat similar to the strategy to be adopted by the HKSAR Government in March, i.e., arranging the testing in the order of the year of birth [23]; (3) Allocating the resources according to the number of confirmed cases detected from each district (case-based strategy); (4) Allocating the resources according to our transmission-guided prioritization strategy, i.e., taking into account the population size, the detected case number of each district, and more importantly, the transmission risk that this district could bring to others.

**Fig. 4:**
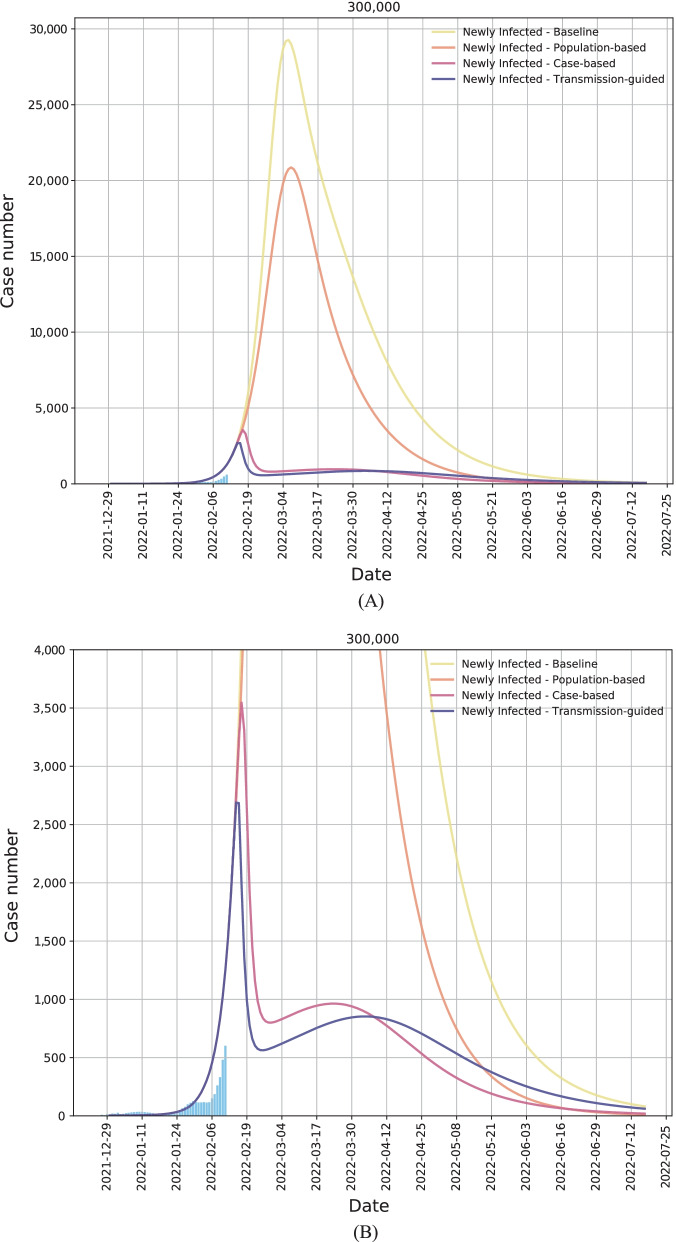

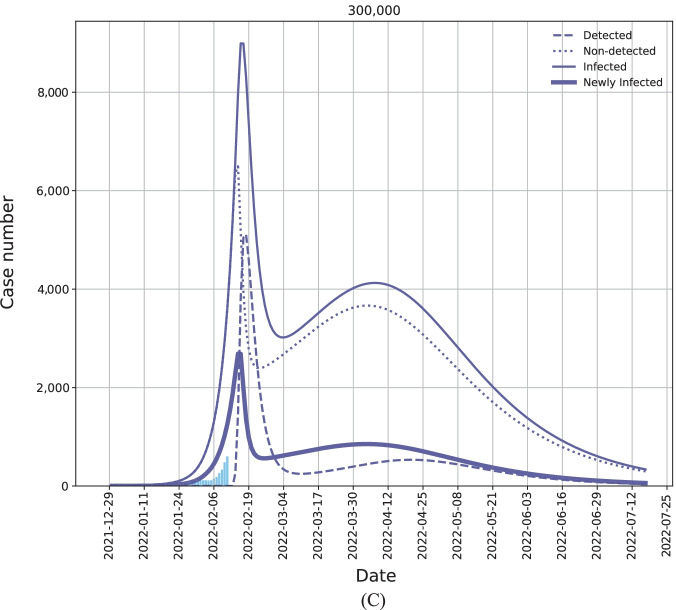
The simulation of the trend of Omicron outbreak (in terms of the daily case number) in Hong Kong, China from December 30, 2021 to July 2022. We assume that the 300,000 testing capacity per day will be available from February 14, 2022. (**A**) Four scenarios with various resource allocation strategies: the baseline (yellow curve), population-based strategy (orange curve), case-based strategy (red curve), and our transmission-guided strategy (blue curve). (**B**) The detailed comparison between the case-based strategy (red curve) and the proposed transmission-guided strategy (blue curve). (**C**) The details of the daily infection trend with our transmission-guided resource allocation. The thicker solid curve denotes the number of daily new infections; the thinner solid curve denotes the total number of infected individuals in each day, including both the newly infected ones and the previously infected but not recovered individuals; the dash line denotes the number of detected cases; and the dotted line denotes the number of non-detected cases

Figure 4A, B shows the simulation results of the aforementioned four scenarios with the capacity of 300,000 testing per day. The simulation starts from December 30, 2021 and it lasts for 200 days. The blue bars show the number of daily confirmed cases with a 7-day moving average effect from December 27, 2021 to February 11, 2022. In our scenario analyses, we start allocating the testing resources from February 14, 2022. The four solid curves with different colors denote the simulation results on the number of daily new infections under the aforementioned four scenarios in the coming 5 months. Please refer to the Additional file 1 for more details of simulation settings.

Compared to the scenarios with the baseline and the population-based strategy (the yellow curve and the orange curve in Fig. 4A, B), our transmission-guided strategy largely flattens the peak of outbreaks in terms of the number of daily new infections. Specifically, compared with the population-based strategy, the proposed transmission-guided strategy can reduce the infection peak by 87.1% (case number reduces from 20 860 to 2689), which can be observed from Fig. 4A. Even compared with the case-based strategy, as enlarged and illustrated in Fig. 4B, our transmission-guided strategy can further reduce the infection peak by 24.2% (case number reduces from 3547 to 2689), demonstrating the efficacy of the developed resource allocation strategy in alleviating the burden of the healthcare system. The reduction percentages of the peaking infections by using transmission-guided allocation strategy compared with other three allocation strategies for all three testing capacity scenarios: 300,000, 500,000, and 700,000 tests per day are provided in Table S4 of the Additional file 1.

We show more details of our transmission-guided strategy with the 300,000 testing capacity in Fig. 4C. The thicker solid curve denotes the number of daily new infections, which is the same as the blue solid curve in Fig. 4A, B. The thinner solid curve denotes the total number of infected individuals in each day, including both the newly infected ones and the previously infected but not recovered individuals. As explained in the Methods Section, we further divide the total number of infected individuals into two sub-groups: the detected infections and non-detected infections, which are shown by the dash line and the dotted line, respectively. From Fig. 4C, we can observe that the number of detected infections will increase rapidly at the beginning. The possible reason is that the appropriately allocated resources make the identification of infections effective, and thus more cases can be detected via the testing. After a few days, this number and the number of total infections (daily) start to decrease, indicating the effectiveness of the developed strategy in controlling the disease spread. However, due to the insufficiency of the testing resources in making a comprehensive testing, some non-detected cases will continue infecting other individuals, resulting in the second round of the case number increasing and peaking.

**Fig. 5:**
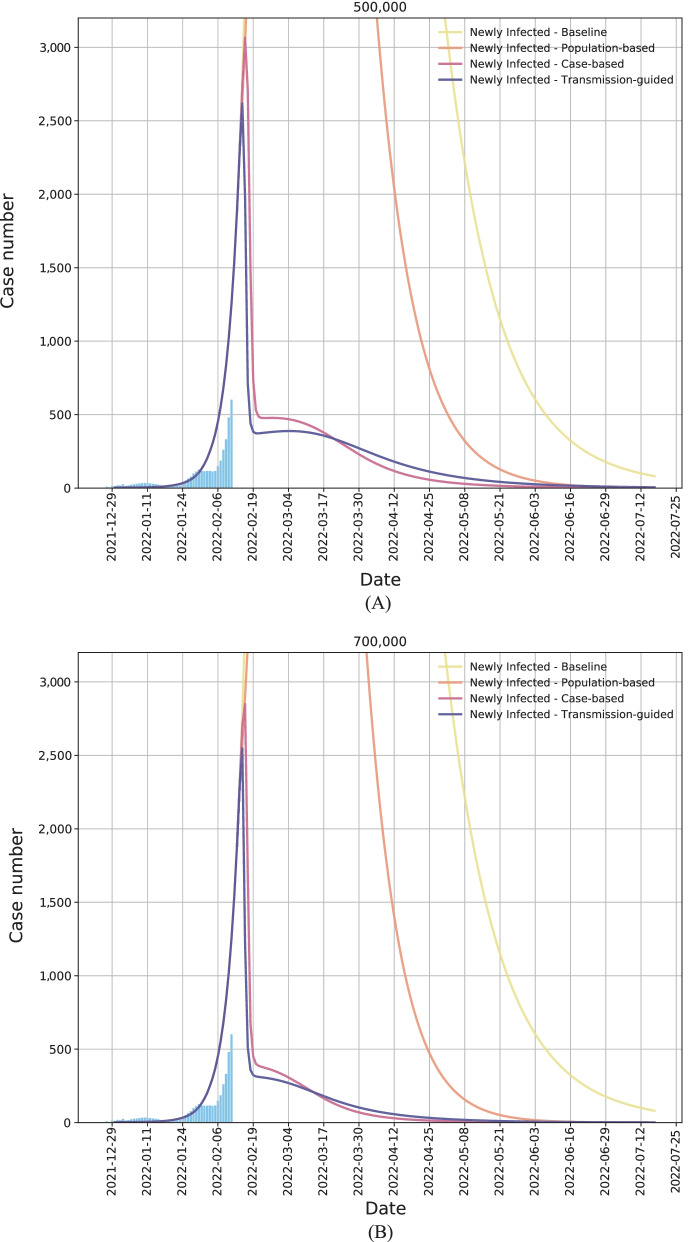
The simulation of the trend of Omicron outbreak (in terms of the daily case number) in Hong Kong, China from December 30, 2021 to July 17, 2022. We assume that (**A**) 500,000 and (**B**) 700,000 testing capacity per day will be available from February 14, 2022

In addition to the simulation with the testing capacity of 300,000 per day, we also demonstrate the simulation results with the testing capacity of 500,000 and 700,000 in Fig. 5A and B, respectively. With more testing resources available, the infection peak can be further flattened, and the outbreak can be alleviated in a more effective way: with both the increased capacity and our transmission-guided resource allocation strategy, the number of daily new infections can be reduced sharply, i.e., from around 2500 to around 300, in less than 5 days. Moreover, the height of the second peak with the 500,000/700,000 capacity will be much lower than that with the 300,000 testing capacity, and the epidemic (in terms of the number of daily new infections) can be quickly under control, with the effective resource prioritization.

## Discussion

The scenario analyses demonstrate that the proposed resource allocation strategy is more effective in alleviating the disease spread than the population-based and case number-based allocation strategies, indicating the necessity and importance of incorporating the spatiotemporal transmission patterns of disease in foreseeing the outbreak risk and allocating the healthcare resources, especially in densely populated regions. The results also imply that only an effective resource allocation strategy might not be sufficient to control the outbreaks, especially when the capacity is extremely limited. With more capacity available, our resource prioritization strategy will be more powerful in containing the disease transmission.

Note that in our method, we emphasize the spatiotemporal transmission because the disease spread is mainly caused by the human mobility across districts over time. This makes the transmission intensity and risk varying over time, in a daily basis. Moreover, we define the daily transmission risk of each district as its overall transmission potential to all 18 districts (including itself). This means that we calculate the risk of each district by summating the out-going transmissibility from this district to others, rather the in-coming transmissibility received from other districts. By doing so, we expect to make the characterized risk more foreseeing, i.e., enabling the quantification that how will this district’s risk impact the disease transmission in other districts. This is consistent with our primary goal of actively seeing the future dynamics of the outbreak and effectively planning for disease control.

The computational method developed in this study is general and can also be used in guiding public-health responses to the potential future outbreaks caused by other new variants in Hong Kong, China as well as in other densely populated countries and regions. However, when applying the developed method to other countries or regions with similar requirements (i.e., how to effectively allocate limited resources for disease control), we need to adaptively implement the country/region-specific settings (e.g., the spatial and/or temporal resolutions of data and model, the ways of constructing spatiotemporal transmission matrices from the individual case information, and the levels of healthcare capacity), rather than directly using the numerical results obtained in this paper.

## Conclusions

In this study, we develop a resource allocation strategy for prioritizing limited healthcare capacity based on the computational characterization of spatiotemporal patterns of the disease transmission risks. Compared with the population-based and case-based strategies, the developed strategy foresees the out-going transmission risks of different districts in a more active way, thus enabling the planning of resource prioritization ahead, which is of critical importance in achieving timely outbreak control under insufficient capacity. Through the comprehensive investigation of risk mapping and resource allocation in Hong Kong, China, we gain the insights into the dynamism and heterogeneity of the outbreaks of Omicron as well as the implementation and optimization of the prioritized resource allocation strategies. As a general approach, the proposed transmission-guided strategy can help guide public-health responses not only to the Omicron outbreaks but also to the potential future outbreaks caused by other new variants. Moreover, the investigation conducted in Hong Kong, China provides useful suggestions on how to achieve effective disease control with insufficient capacity in other highly populated countries and regions.

## Supplementary Information

**Additional file 1.** Model details, experimental settings, and supporting data.

## Data Availability

All codes and data will be available from the authors upon reasonable request.
