# Supplementary material for: Optimal resource allocation with spatiotemporal transmission discovery for effective disease control
Source: Infect Dis Poverty. 2022 Mar 25;11:34. doi: 10.1186/s40249-022-00957-1 (PMC8947954; doi:10.1186/s40249-022-00957-1)
Supplement: Supplementary file 1 — Additional file 1. Model details, experimental settings, and supporting data. [file 40249_2022_957_MOESM1_ESM.docx]

**Supplementary Material**

**Methods**

**Construction of spatiotemporal transmission matrices**

We build the disease transmission matrices in Hong Kong by considering the spatiotemporal information. According to the visiting records of confirmed cases, we calculate the disease transmission intensity from location $i$ to $j$ brought by a single case using the following equation:

$$\begin{aligned} {st}_{ij}=Dist(D_{v}-D_{o}),\#\left( AUTONUMLGL \backslash* Arabic \backslash e \right) \end{aligned}$$

where ${st}_{ij}$ represents the transmission intensity of one case from location $i$ to $j$, $Dist(\cdot)$ denotes the infectiousness distribution of confirmed cases in terms of the days before and after symptom onset [S1], $D_{o}$ is the onset date, and $D_{v}$ is the visiting date from location $i$ to location $j$. If the case is asymptomatic, there are no records of the onset date, so we use the serial interval distribution to characterize its infectiousness, which is a function of the range of time between the symptom onset of consecutive cases in the chains of transmission and set the report day as the onset day. Based on the definition of infectiousness of a single case from one location to another, we expand the calculation to the situation of multiple cases to measure the overall situation. For all confirmed cases from locations $i$ to $j$, the transmission intensity is calculated as:

$$\begin{aligned} {ST}_{ij}=1-\prod_{n=1}^{N_{ij}} \left( {1-Dist\left( D_{v}-D_{o} \right)}_{n} \right),\#\left( AUTONUMLGL \backslash* Arabic \backslash e \right) \end{aligned}$$

where $N_{ij}$ is the number of cases from location $i$ to $j$, and ${ST}_{ij}$ represents the transmission intensity of these cases from location $i$ to $j$. We construct the spatiotemporal transmission matrix with two steps: (1) within-district construction (constituency-level) and (2) between-district construction (district-level). In the first step, we calculate the within-district disease transmission matrix, the size of which is 452 $\times$ 452. In the second step, the 452 $\times$ 452 constituency-level matrix will be aggregated to an $18\times18$ district-level matrix by summating all elements in the corresponding block of the specific districts.

**District-level risk mapping**

Let the scalar $y_{\left[ t+1 \right]}$ denote the overall transmission intensity from a district to others at the time step $t+1$ (i.e., the next day), $\boldsymbol{x}_{\left[ t-3:t \right]}$ denote the vector composed of previous three days’ transmission intensity, and $f\left( \cdot\right)$ denote the mapping function to project the intensity of the previous three days to that of the next day. Then we have $y_{\left[ t+1 \right]}=f\left( \boldsymbol{x}_{\left[ t-3:t \right]} \right)$. For simplicity, we omit the subscript and let $\boldsymbol{X \in}\mathbb{R}^{\boldsymbol{N\times3}}$ and $\mathbf{y}\boldsymbol{\in}\mathbb{R}^{\boldsymbol{N\times3}}$ be the sample matrix and the label vector of the training data, respectively. In the Gaussian process regression, we have $f\left( \cdot\right)\sim N(\mu,K)$, where $\mu,K$ are the mean function and the kernel function, respectively, which define a Gaussian process. For $\boldsymbol{x}_{i}, \boldsymbol{x}_{j} \in\boldsymbol{X}$, we have:

$$\begin{aligned} \mu\left( \boldsymbol{x}_{i} \right)=E\left( \boldsymbol{x}_{i} \right),\#\left( AUTONUMLGL \backslash* Arabic \backslash e \right) \end{aligned}$$

$$\begin{aligned} k_{ij}\left( \boldsymbol{x}_{i}, \boldsymbol{x}_{j} \right)=E\left( \left( f\left( \boldsymbol{x}_{i} \right)-\mu\left( \boldsymbol{x}_{i} \right) \right)\left( f\left( \boldsymbol{x}_{j} \right)-\mu\left( \boldsymbol{x}_{j} \right) \right)^{T} \right).\#\left( AUTONUMLGL \backslash* Arabic \backslash e \right) \end{aligned}$$

Commonly, the mean function will be set to zero, and here we use the linear kernel $k_{Linear}\left( \boldsymbol{x}, \boldsymbol{x}^{*} \right)=\boldsymbol{x}^{T}\boldsymbol{x}^{*}$. According to the property of gaussian distribution, $y^{*}$ (i.e., the prediction of $\boldsymbol{x}^{*}$) will follow a multivariate distribution with the training set and can be written as:

$$\left[ \begin{matrix} f(\boldsymbol{X}) \\ y^{*} \end{matrix} \right]\sim N(\mathbf{0},\left[ \begin{matrix} \boldsymbol{K}(\boldsymbol{X},\boldsymbol{X}) & \boldsymbol{K}(\boldsymbol{X},\boldsymbol{x}^{*}) \\ \boldsymbol{K}(\boldsymbol{x}^{*},\boldsymbol{X}) & \boldsymbol{K}(\boldsymbol{x}^{*},\boldsymbol{x}^{*}) \end{matrix} \right]),$$

According to [S2], we have:

$$\begin{aligned} y^{*}|\boldsymbol{x}^{*},\boldsymbol{X,}\mathbf{y}\sim N(\mu^{\boldsymbol{*}}\boldsymbol{,}\boldsymbol{\Sigma}^{\boldsymbol{*}}\boldsymbol{)},\#\left( 5 \right) \end{aligned}$$

where ${\mu^{\boldsymbol{*}}\boldsymbol{=K}\left( \boldsymbol{x}^{*},\boldsymbol{X} \right)}^{T}{\boldsymbol{K}(\boldsymbol{X},\boldsymbol{X})}^{-1}\mathbf{y}$ and $\boldsymbol{\Sigma}^{\boldsymbol{*}}\boldsymbol{=}$ $\boldsymbol{K}(\boldsymbol{x}^{*},\boldsymbol{x}^{*})-\boldsymbol{K}(\boldsymbol{x}^{*},\boldsymbol{X}){\boldsymbol{K}(\boldsymbol{X},\boldsymbol{X})}^{-1}\boldsymbol{K}(\boldsymbol{X},\boldsymbol{x}^{*})$. The $\mu^{\boldsymbol{*}}$ in the above equation will be used as the prediction of $y^{*}$.

**Simulation of resource allocation**

We develop our Susceptible-Detected-Nondetected-Recovered (SDNR) compartmental model at the meta-population level by dividing the infection status in original SIR model into two sub-groups: detected and non-detected infections, with different recovery rates. We introduce a parameter, called allocation rate, to depict the transition from detected infections to non-detected infections. Furthermore, to describe the disease transmission process between different districts, we incorporate the risk transmission matrix $T$ to depict the transmission contacts between the individuals from different districts. The differential equations of our model are given as follows:

$$\begin{aligned} \left\{ \begin{aligned} &\frac{dS_{i}\left( t \right)}{dt}=-\beta_{i}\left( \alpha_{i}T_{i,i}I_{ND,i}\left( t \right)+\sum_{j=1,j\neq i}^{N} \alpha_{j}T_{i,j}I_{ND,j} \right)S_{i}\left( t \right) \\ &\frac{dI_{D,i}\left( t \right)}{dt}=C_{i,i}I_{ND,i}\left( t \right)-\gamma_{2,i}I_{D,i}\left( t \right) \\ &\frac{dI_{ND,i}\left( t \right)}{dt}=\beta_{i}\left( \alpha_{i}T_{i,i}I_{ND,i}\left( t \right)+\sum_{j=1,j\neq i}^{N} \alpha_{j}T_{i,j}I_{ND,j} \right)S_{i}\left( t \right)-C_{i,i}I_{ND,i}\left( t \right)-\gamma_{1,i}I_{ND,i}\left( t \right) \\ &\frac{dR_{i}\left( t \right)}{dt}=\gamma_{2,i}I_{D,i}\left( t \right)+\gamma_{1,i}I_{ND,i}\left( t \right) \end{aligned}, \right.\#\left( AUTONUMLGL \backslash* Arabic \backslash e \right) \end{aligned}$$

where $S_{i}\left( t \right), I_{D,i}\left( t \right), I_{ND,i}\left( t \right), R_{i}\left( t \right)$ represent the susceptible individuals, detected infections, non-detected infections, and recovered individuals in the $i$-th district at time *t*, respectively. Parameters $\alpha_{i}, \beta_{i}, \gamma_{1,i}, \gamma_{2,i}$ are the infectious rate, susceptible rate, recovery rate of non-detected individuals and recovery rate of detected individuals, respectively. The $C_{i,i}$ is the allocation rate of the testing ability for the $i$-th district, which is the ratio of the allocated testing resources to the population size in the $i$-th district. This allocation rate denotes the proportion of infected individuals transiting from the non-detected status to the detected status. The $T_{i,j}$ denotes the transmission risk from the $i$-th district to $j$-th district, and $T_{i,i}$ denotes the transmission risk within the $i$-th district. For all districts, these parameters can be represented in diagonal matrices $A, B,$ $\boldsymbol{\gamma}_{1}$, $\boldsymbol{\gamma}_{2}$, and $C$, respectively. Following the construction of next-generation matrix in [S3], we can construct the matrix $\boldsymbol{K}$, which denotes the new non-detected infections brought by previous non-detected infected individuals in all districts:

$$\begin{aligned} \boldsymbol{K}=SB\left( \boldsymbol{\gamma}_{1}+C \right)^{-1}T^{T}A,\#\left( AUTONUMLGL \backslash* Arabic \backslash e \right) \end{aligned}$$

where $S$ is a diagonal matrix, with its diagonal elements $S_{i,i}$ denoting the proportion of susceptible individuals to the population in the $i$-th district. Correspondingly, its effective reproduction number $R_{t}$ can be represented as the largest eigenvalue of the next-generation matrix:

$$\begin{aligned} R_{t}=\rho\left( \boldsymbol{K} \right)=\rho\left( SB\left( \boldsymbol{\gamma}_{1}+C \right)^{-1}T^{T}A \right),\#\left( AUTONUMLGL \backslash* Arabic \backslash e \right) \end{aligned}$$

where $\rho\left( \cdot\right)$ represents the eigenvalue decomposition.

We start our simulation from December 30, 2021, which is the beginning of the Omicron outbreak in Hong Kong, China. We set the parameter $\alpha_{i}, \beta_{i}, \gamma_{1,i}, \gamma_{2,i}$ (*i* = 1, …, 18, and the code and name of the 18 districts are provided in Table S1) as the same for all districts and parameterize the risk transmission matrix $T$ by the constructed transmission matrices based on the several COVID-19 outbreaks from March 17, 2020, to February 5, 2022. Specifically, we set $\alpha_{i}=0.29$, $\beta_{i}=0.8$, $\gamma_{1,i}=0.2$, $\gamma_{1,i}=0.25$, and at the start point of the simulation, we set the $S, I_{D}, I_{ND},R$ based on the confirmed case number and the population in different districts on December 29, 2021, and we assume that the number of non-detected infections is the same as that of confirmed cases at the starting point of our simulation. Since there is a long zero-case stage in Hong Kong, China before the Omicron outbreak, we construct daily transmission matrices based on the three outbreaks during the period of March 17, 2020 to February 14, 2021, and take the average of them as the historical transmission patterns. We construct daily transmission matrices based on the latest data of the Omicron outbreak from December 27, 2021 to February 05, 2022, and calculate a 4-day moving average as the transmission patterns for that day (e.g., the transmission pattern for December 30, 2021 is the average of the four matrices from December 27, 2021 to December 30, 2021). Then we calculate the final transmission matrix in each day from December 30, 2021 to February 05, 2022 by combining the historical transmission patterns and the latest transmission patterns in a weighted manner to reduce the large fluctuations which are caused by the noise of data.

Table S1. The code and name of 18 districts in Hong Kong, China.

| **District Code** | **District Name** |
| --- | --- |
| D1 | Central |
| D2 | Wan Chai |
| D3 | Eastern |
| D4 | Southern |
| D5 | Yau Tsim Mong |
| D6 | Sham Shui Po |
| D7 | Kowloon City |
| D8 | Wong Tai Sin |
| D9 | Kwun Tong |
| D10 | Tsuen Wan |
| D11 | Tuen Mun |
| D12 | Yuen Long |
| D13 | North |
| D14 | Tai Po |
| D15 | Sai Kung |
| D16 | Shatin |
| D17 | Kwai Tsing |
| D18 | Islands |

As mentioned in the Methods Sections in the main body of our paper, we examine four different scenarios with various testing resource allocation strategies under three levels of testing capacity, which are assumed to be available from February 14, 2022. Before this date, we assume no specific allocation strategy was adopted, that means we set the $C$ as a zero matrix. Stating from February 14, 2022, we set the specific allocation rates as follow: (1) Baseline: we set the allocation rate for all districts to be zero. (2) Population-based allocation: we set the allocation proportion of testing resources for the $i$-th district as $C_{i,i}=\frac{{population}_{i}}{{population}_{total}}\cdot\frac{allocation capacity}{{population}_{i}}$. (3) Case-based allocation: we set the allocation rate as $C_{i,i}=\frac{{cumulative newly detected case}_{i}}{{cumulative newly detected case}_{total}}\cdot\frac{allocation capacity}{{population}_{i}}$. (4) Transmission-guided allocation: we set the allocation rate as $C_{i,i}=normalize\left( \frac{{population}_{i}}{{population}_{total}}\cdot\frac{{cumulative newly detected case}_{i}}{{cumulative newly detected case}_{total}}\cdot\frac{\sum_{j=1}^{N} T_{i,j}}{\sum_{i=1}^{N} \sum_{j=1}^{N} T_{i,j}} \right)\cdot\frac{allocation capacity}{{population}_{i}}$.

**Results**

**Results of the transmission matrix construction**

The details of the constructed transmission matrices on January 20, 2022, and January 31, 2022 are provided in the Table S2 and Table S3 respectively.

Table S2. The constructed transmission matrix on January 20, 2022

|  | **D1** | **D2** | **D3** | **D4** | **D5** | **D6** | **D7** | **D8** | **D9** | **D10** | **D11** | **D12** | **D13** | **D14** | **D15** | **D16** | **D17** | **D18** |
| --- | --- | --- | --- | --- | --- | --- | --- | --- | --- | --- | --- | --- | --- | --- | --- | --- | --- | --- |
| **D1** | 0.97 | 0.04 | 0.00 | 0.00 | 0.12 | 0.00 | 0.00 | 0.00 | 0.00 | 0.00 | 0.00 | 0.00 | 0.00 | 0.00 | 0.00 | 0.00 | 0.00 | 0.00 |
| **D2** | 0.12 | 0.06 | 0.00 | 0.00 | 0.12 | 0.00 | 0.00 | 0.00 | 0.00 | 0.00 | 0.00 | 0.00 | 0.00 | 0.00 | 0.00 | 0.00 | 0.00 | 0.00 |
| **D3** | 0.00 | 0.00 | 0.08 | 0.00 | 0.00 | 0.00 | 0.00 | 0.00 | 0.00 | 0.00 | 0.00 | 0.00 | 0.00 | 0.00 | 0.00 | 0.00 | 0.00 | 0.00 |
| **D4** | 0.00 | 0.00 | 0.00 | 0.65 | 0.00 | 0.00 | 0.00 | 0.00 | 0.00 | 0.00 | 0.00 | 0.00 | 0.00 | 0.00 | 0.00 | 0.00 | 0.00 | 0.00 |
| **D5** | 0.12 | 0.12 | 0.00 | 0.00 | 0.72 | 0.00 | 0.00 | 0.00 | 0.00 | 0.04 | 0.00 | 0.00 | 0.00 | 0.00 | 0.00 | 0.00 | 0.00 | 0.00 |
| **D6** | 0.00 | 0.00 | 0.00 | 0.00 | 0.00 | 0.07 | 0.00 | 0.00 | 0.00 | 0.00 | 0.00 | 0.00 | 0.00 | 0.00 | 0.00 | 0.00 | 0.00 | 0.00 |
| **D7** | 0.00 | 0.00 | 0.00 | 0.00 | 0.06 | 0.00 | 0.07 | 0.00 | 0.00 | 0.00 | 0.00 | 0.00 | 0.00 | 0.00 | 0.00 | 0.00 | 0.04 | 0.00 |
| **D8** | 0.00 | 0.00 | 0.00 | 0.00 | 0.04 | 0.00 | 0.00 | 0.03 | 0.00 | 0.00 | 0.00 | 0.00 | 0.00 | 0.00 | 0.00 | 0.00 | 0.00 | 0.00 |
| **D9** | 0.00 | 0.00 | 0.00 | 0.00 | 0.00 | 0.00 | 0.00 | 0.00 | 0.03 | 0.00 | 0.00 | 0.00 | 0.00 | 0.00 | 0.00 | 0.00 | 0.00 | 0.00 |
| **D10** | 0.00 | 0.00 | 0.00 | 0.00 | 0.00 | 0.00 | 0.00 | 0.00 | 0.02 | 0.49 | 0.12 | 0.00 | 0.00 | 0.00 | 0.00 | 0.00 | 0.03 | 0.00 |
| **D11** | 0.00 | 0.00 | 0.00 | 0.00 | 0.00 | 0.00 | 0.00 | 0.00 | 0.00 | 0.00 | 0.00 | 0.00 | 0.00 | 0.00 | 0.00 | 0.00 | 0.00 | 0.00 |
| **D12** | 0.00 | 0.00 | 0.00 | 0.00 | 0.00 | 0.00 | 0.00 | 0.00 | 0.00 | 0.00 | 0.00 | 0.00 | 0.00 | 0.00 | 0.00 | 0.00 | 0.00 | 0.00 |
| **D13** | 0.00 | 0.00 | 0.00 | 0.00 | 0.00 | 0.00 | 0.00 | 0.00 | 0.00 | 0.03 | 0.00 | 0.00 | 0.01 | 0.00 | 0.00 | 0.01 | 0.00 | 0.00 |
| **D14** | 0.00 | 0.00 | 0.00 | 0.00 | 0.00 | 0.00 | 0.00 | 0.00 | 0.00 | 0.00 | 0.00 | 0.00 | 0.00 | 0.00 | 0.00 | 0.00 | 0.00 | 0.00 |
| **D15** | 0.00 | 0.00 | 0.00 | 0.00 | 0.00 | 0.00 | 0.00 | 0.00 | 0.00 | 0.00 | 0.00 | 0.00 | 0.00 | 0.00 | 0.00 | 0.00 | 0.00 | 0.00 |
| **D16** | 0.00 | 0.00 | 0.00 | 0.00 | 0.00 | 0.00 | 0.00 | 0.00 | 0.02 | 0.03 | 0.00 | 0.00 | 0.00 | 0.00 | 0.00 | 0.09 | 0.00 | 0.00 |
| **D17** | 0.00 | 0.00 | 0.00 | 0.00 | 0.00 | 0.00 | 0.00 | 0.00 | 0.00 | 1.15 | 0.00 | 0.00 | 0.00 | 0.00 | 0.00 | 0.00 | 1.64 | 0.00 |
| **D18** | 0.00 | 0.00 | 0.00 | 0.00 | 0.00 | 0.00 | 0.00 | 0.00 | 0.00 | 0.00 | 0.00 | 0.00 | 0.00 | 0.00 | 0.00 | 0.00 | 0.00 | 0.19 |

Table S3. The constructed transmission matrix on January 31, 2022

|  | **D1** | **D2** | **D3** | **D4** | **D5** | **D6** | **D7** | **D8** | **D9** | **D10** | **D11** | **D12** | **D13** | **D14** | **D15** | **D16** | **D17** | **D18** |
| --- | --- | --- | --- | --- | --- | --- | --- | --- | --- | --- | --- | --- | --- | --- | --- | --- | --- | --- |
| **D1** | 0.34 | 0.08 | 0.00 | 0.06 | 0.18 | 0.00 | 0.00 | 0.00 | 0.00 | 0.00 | 0.00 | 0.22 | 0.00 | 0.00 | 0.00 | 0.16 | 0.00 | 0.00 |
| **D2** | 0.16 | 1.59 | 0.00 | 0.00 | 0.24 | 0.00 | 0.00 | 0.04 | 0.00 | 0.00 | 0.00 | 0.52 | 0.00 | 0.00 | 0.00 | 0.68 | 0.00 | 0.04 |
| **D3** | 0.00 | 0.00 | 0.04 | 0.00 | 0.00 | 0.00 | 0.06 | 0.00 | 0.00 | 0.00 | 0.00 | 0.00 | 0.00 | 0.00 | 0.00 | 0.00 | 0.00 | 0.00 |
| **D4** | 0.00 | 0.00 | 0.00 | 0.00 | 0.00 | 0.00 | 0.00 | 0.00 | 0.00 | 0.00 | 0.00 | 0.00 | 0.00 | 0.00 | 0.00 | 0.00 | 0.00 | 0.00 |
| **D5** | 0.00 | 0.00 | 0.00 | 0.00 | 1.69 | 0.00 | 0.00 | 0.00 | 0.00 | 0.00 | 0.08 | 0.00 | 0.00 | 0.00 | 0.00 | 0.15 | 0.04 | 0.00 |
| **D6** | 0.00 | 0.00 | 0.00 | 0.00 | 0.00 | 1.68 | 0.00 | 0.00 | 0.00 | 0.00 | 0.00 | 0.01 | 0.00 | 0.00 | 0.00 | 0.00 | 0.00 | 0.00 |
| **D7** | 0.00 | 0.00 | 0.00 | 0.00 | 0.00 | 0.00 | 0.54 | 0.00 | 0.00 | 0.00 | 0.00 | 0.00 | 0.00 | 0.00 | 0.00 | 0.12 | 0.00 | 0.00 |
| **D8** | 0.00 | 0.13 | 0.00 | 0.00 | 0.00 | 0.00 | 0.00 | 7.59 | 0.37 | 0.00 | 0.00 | 0.00 | 0.00 | 0.39 | 0.00 | 0.00 | 0.00 | 0.00 |
| **D9** | 0.00 | 0.00 | 0.00 | 0.00 | 0.00 | 0.00 | 0.04 | 0.02 | 1.92 | 0.00 | 2.06 | 0.00 | 0.00 | 0.00 | 0.00 | 0.00 | 0.00 | 0.00 |
| **D10** | 0.00 | 0.00 | 0.00 | 0.00 | 0.00 | 0.00 | 0.00 | 0.00 | 0.00 | 0.41 | 0.00 | 0.00 | 0.00 | 0.00 | 0.00 | 0.12 | 0.00 | 0.00 |
| **D11** | 0.00 | 0.00 | 0.00 | 0.00 | 0.08 | 0.00 | 0.00 | 0.00 | 0.31 | 0.00 | 2.15 | 0.00 | 0.00 | 0.00 | 0.00 | 0.00 | 0.08 | 0.00 |
| **D12** | 0.20 | 0.06 | 0.00 | 0.00 | 0.00 | 0.01 | 0.00 | 0.00 | 0.00 | 0.00 | 0.00 | 1.23 | 0.00 | 0.00 | 0.00 | 0.12 | 0.00 | 0.00 |
| **D13** | 0.00 | 0.00 | 0.00 | 0.00 | 0.00 | 0.00 | 0.00 | 0.00 | 0.00 | 0.00 | 0.00 | 0.00 | 0.04 | 0.00 | 0.00 | 0.00 | 0.00 | 0.00 |
| **D14** | 0.00 | 0.00 | 0.00 | 0.00 | 0.69 | 0.00 | 0.00 | 0.13 | 0.00 | 0.00 | 0.00 | 0.00 | 0.00 | 2.72 | 0.00 | 0.00 | 0.00 | 0.00 |
| **D15** | 0.00 | 0.00 | 0.00 | 0.00 | 0.00 | 0.00 | 0.00 | 0.00 | 0.00 | 0.00 | 0.00 | 0.00 | 0.00 | 0.00 | 0.00 | 0.00 | 0.00 | 0.00 |
| **D16** | 0.32 | 0.08 | 0.00 | 0.00 | 0.00 | 0.16 | 0.00 | 0.00 | 0.00 | 0.08 | 0.00 | 0.44 | 0.00 | 0.00 | 0.00 | 6.60 | 0.00 | 0.00 |
| **D17** | 0.00 | 0.00 | 0.00 | 0.00 | 0.04 | 0.00 | 0.00 | 0.00 | 0.00 | 0.16 | 0.00 | 0.00 | 0.00 | 0.00 | 0.00 | 0.00 | 13.51 | 0.00 |
| **D18** | 0.00 | 0.00 | 0.00 | 0.00 | 0.02 | 0.00 | 0.00 | 0.00 | 0.00 | 0.00 | 0.00 | 0.00 | 0.00 | 0.00 | 0.00 | 0.00 | 0.00 | 0.06 |

**District-level risk mapping**

The details of the risk mapping results of 18 districts are provided in Table S5. The details of the risk ranking results of 18 districts are provided in Table S6.

**Scenario analyses of transmission-guided resource allocation**

Table S4 demonstrates the reduction percentage of the peaking infections using transmission-guided allocation strategy compared with other three allocation strategies in three testing capacity scenarios, i.e. 300,000, 500,000, and 700,000 tests per day.

Table S4. The reduction percentage of the peaking infections using transmission-guided allocation strategy compared with other three allocation strategies in three testing capacity scenarios: 300,000, 500,000, and 700,000 tests per day.

| **Allocation Strategy** | **300,000** | **500,000** | **700,000** |
| --- | --- | --- | --- |
| Baseline | 90.8% (from 29,264 to 2,689) | 91.1% (from 29,264 to 2,617) | 91.3% (from 29,264 to 2,546) |
| Population-based | 87.1% (from 20,860 to 2,689) | 83.6% (from 15,950 to 2,617) | 78.2% (from 11,693 to 2,546) |
| Case-based | 24.2% (from 3,547 to 2,689) | 14.6% (from 15,950 to 2,617) | 10.6% (from 2,849 to 2,546) |

**Software and packages**

Our method and simulations are implemented by Python 3. The packages used to plot figures include GeoPandas, NetworkX, and Matplotlib.

**Supplementary References**

1. He X, Lau EH, Wu P, Deng X, Wang J, Hao X, et al. Temporal dynamics in viral shedding and transmissibility of COVID-19. *Nat Med*. 2020 May;26(5):672-5.
2. Williams CK. CE Rasmussen Gaussian Processes for Regression. *NIPS*. 1996;8.
3. Diekmann O, Heesterbeek JA, Roberts MG. The construction of next-generation matrices for compartmental epidemic models. *J R Soc Interface*. 2010 Jun 6;7(47):873-85.

Table S5. The risk mapping of 18 districts in Hong Kong, China from January 30, 2022 to February 5, 2022.

| Disrtict | January 30, 2022 | January 31, 2022 | February 1, 2022 | February 2, 2022 | February 3, 2022 | February 4, 2022 | February 5, 2022 |
| --- | --- | --- | --- | --- | --- | --- | --- |
| Central | 0.03 | 0.00 | 0.22 | 0.15 | 0.12 | 0.00 | 0.00 |
| Wan Chai | 0.12 | 0.04 | 0.04 | 0.26 | 0.13 | 0.02 | 0.00 |
| Eastern | 0.03 | 0.04 | 0.02 | 0.01 | 0.05 | 0.00 | 0.00 |
| Southern | 0.00 | 0.00 | 0.00 | 0.00 | 0.04 | 0.03 | 0.04 |
| Yau Tsim Mong | 0.13 | 0.50 | 0.26 | 0.16 | 0.00 | 0.06 | 0.00 |
| Sham Shui Po | 0.15 | 0.13 | 0.27 | 0.14 | 0.17 | 0.19 | 0.15 |
| Kowloon City | 0.03 | 0.06 | 0.00 | 0.08 | 0.05 | 0.34 | 0.22 |
| Wong Tai Sin | 0.35 | 0.32 | 0.71 | 0.77 | 0.40 | 0.00 | 0.00 |
| Kwun Tong | 0.05 | 0.00 | 0.12 | 0.40 | 0.30 | 0.08 | 0.00 |
| Tsuen Wan | 0.00 | 2.20 | 0.78 | 0.45 | 0.00 | 0.00 | 1.00 |
| Tuen Mun | 0.00 | 0.20 | 0.22 | 0.21 | 0.00 | 0.26 | 2.55 |
| Yuen Long | 0.03 | 0.29 | 0.24 | 0.22 | 0.96 | 0.29 | 0.37 |
| North | 0.01 | 0.03 | 0.02 | 0.00 | 0.00 | 0.01 | 0.00 |
| Tai Po | 0.10 | 0.03 | 0.00 | 0.33 | 0.22 | 0.21 | 0.00 |
| Sai Kung | 0.01 | 0.00 | 0.02 | 0.00 | 0.03 | 0.00 | 0.00 |
| Shatin | 0.13 | 0.59 | 0.30 | 0.86 | 0.63 | 0.28 | 0.00 |
| Kwai Tsing | 0.00 | 0.00 | 0.00 | 1.40 | 0.00 | 0.22 | 0.00 |
| Islands | 0.00 | 0.00 | 0.05 | 0.00 | 0.00 | 0.16 | 0.11 |

Table S6. The risk ranking results of 18 districts in Hong Kong, China from January 30, 2022 to February 5, 2022.

| Risk Ranking | January 30, 2022 | January 31, 2022 | February 1, 2022 | February 2, 2022 | February 3, 2022 | February 4, 2022 | February 5, 2022 |
| --- | --- | --- | --- | --- | --- | --- | --- |
| 1 | Wong Tai Sin | Tsuen Wan | Tsuen Wan | Kwai Tsing | Yuen Long | Kowloon City | Tuen Mun |
| 2 | Sham Shui Po | Shatin | Wong Tai Sin | Shatin | Shatin | Yuen Long | Tsuen Wan |
| 3 | Yau Tsim Mong | Yau Tsim Mong | Shatin | Wong Tai Sin | Wong Tai Sin | Shatin | Yuen Long |
| 4 | Shatin | Wong Tai Sin | Sham Shui Po | Tsuen Wan | Kwun Tong | Tuen Mun | Kowloon City |
| 5 | Wan Chai | Yuen Long | Yau Tsim Mong | Kwun Tong | Tai Po | Kwai Tsing | Sham Shui Po |
| 6 | Tai Po | Tuen Mun | Yuen Long | Tai Po | Sham Shui Po | Tai Po | Islands |
| 7 | Kwun Tong | Sham Shui Po | Central | Wan Chai | Wan Chai | Sham Shui Po | Southern |
| 8 | Central | Kowloon City | Tuen Mun | Yuen Long | Central | Islands | Eastern |
| 9 | Kowloon City | Wan Chai | Kwun Tong | Tuen Mun | Kowloon City | Kwun Tong | North |
| 10 | Eastern | Eastern | Islands | Yau Tsim Mong | Eastern | Yau Tsim Mong | Sai Kung |
| 11 | Yuen Long | Tai Po | Wan Chai | Central | Southern | Southern | Central |
| 12 | North | North | North | Sham Shui Po | Sai Kung | Wan Chai | Wan Chai |
| 13 | Sai Kung | Sai Kung | Sai Kung | Kowloon City | Yau Tsim Mong | North | Yau Tsim Mong |
| 14 | Southern | Central | Eastern | Eastern | Tsuen Wan | Central | Wong Tai Sin |
| 15 | Tsuen Wan | Southern | Southern | North | Tuen Mun | Eastern | Kwun Tong |
| 16 | Tuen Mun | Kwun Tong | Kowloon City | Southern | North | Wong Tai Sin | Tai Po |
| 17 | Kwai Tsing | Kwai Tsing | Tai Po | Sai Kung | Kwai Tsing | Tsuen Wan | Shatin |
| 18 | Islands | Islands | Kwai Tsing | Islands | Islands | Sai Kung | Kwai Tsing |
